# Association of maximal stress ergometry performance with troponin T and abdominal aortic calcification score in advanced chronic kidney disease

**DOI:** 10.1186/s12882-021-02251-y

**Published:** 2021-02-04

**Authors:** Roosa Lankinen, Markus Hakamäki, Kaj Metsärinne, Niina Koivuviita, Jussi P. Pärkkä, Maria Saarenhovi, Tapio Hellman, Mikko J. Järvisalo

**Affiliations:** 1 Kidney Centre, Turku University Hospital and University of Turku, Turku, Finland; 2 Department of Clinical Physiology and Nuclear Medicine, Turku University Hospital and University of Turku, Turku, Finland; 3 Department of Anaesthesiology and Intensive Care, University of Turku, Turku, Finland; 4 Perioperative Services, Intensive Care and Pain Medicine, Turku University Hospital, Building 18, TG3B, Hämeentie 11, FIN-20520 Turku, Finland

**Keywords:** Chronic kidney disease, Ergometry stress test, Aortic calcification, Troponin T, Echocardiography

## Abstract

**Background:**

Cardiac biomarkers Troponin T (TnT) and N-terminal pro-B-type natriuretic peptide (proBNP) and abdominal aortic calcification score (AAC) are associated with cardiovascular events and mortality in patients with chronic kidney disease (CKD). The effects of cardiac biomarkers and AAC on maximal exercise capacity in CKD are unknown and were studied.

**Methods:**

One hundred seventy-four CKD 4–5 patients not on maintenance dialysis underwent maximal bicycle ergometry stress testing, lateral lumbar radiograph to study AAC, echocardiography and biochemical assessments.

**Results:**

The subjects with proportional maximal ergometry workload (WMAX%) less than 50% of the expected values had higher TnT, proBNP, AAC, left ventricular end-diastolic diameter, left ventricular mass index, E/e’ and pulse pressure, and lower global longitudinal strain compared to the better performing patients. TnT (β = − 0.09, *p* = 0.02), AAC (β = − 1.67, *p* < 0.0001) and diabetes (β = − 11.7, *p* < 0.0001) remained significantly associated with WMAX% in the multivariable model. Maximal ergometry workload (in Watts) was similarly associated with TnT and AAC in addition to age, male gender, hemoglobin and diastolic blood pressure in a respective multivariate model.

AAC and TnT showed fair predictive power for WMAX% less than 50% of the expected value with AUCs of 0.70 and 0.75, respectively.

**Conclusions:**

TnT and AAC are independently associated with maximal ergometry stress test workload in patients with advanced CKD.

**Trial registration:**

http://www.ClinicalTrials.gov NCT04223726.

**Supplementary Information:**

The online version contains supplementary material available at 10.1186/s12882-021-02251-y.

## Background

Cardiovascular disease is the prevailing cause of morbidity and mortality in patients with chronic kidney disease (CKD). Alterations in cardiac and vascular structure and function may lead to exercise intolerance. Impairment of maximal exercise performance is well-established in CKD [1–3], but less is known about its determinants.

Elevated troponin T has been shown to be associated with increased cardiovascular mortality in end stage kidney disease (ESKD) [4–6]. The Food and Drug Administration has approved the use of troponin T (TnT) for identifying high mortality risk in CKD patients [5]. N-terminal pro-B-type natriuretic peptide (proBNP) elevation may occur in CKD independent of heart failure [4], but usually reflects underlying heart disease such as left ventricular hypertrophy or coronary atherosclerosis [7, 8]. proBNP has been associated with cardiovascular events and cardiovascular mortality in CKD [9]. However, there are no previous data on the association between cardiac biomarkers and maximal physical performance in advanced CKD.

Current guidelines recommend the evaluation of abdominal aortic calcification (AAC) score in CKD [10]. AAC is independently associated with cardiovascular events in the general population and in dialysis patients [11, 12]. Previous studies have shown that AAC is independently associated with coronary artery calcification [13], left ventricular diastolic dysfunction [14], and increased risk for incident claudication [15], all of which may lead to diminished physical performance in CKD.

Deteriorating exercise capacity has great importance on the quality of life, morbidity and mortality. Evaluating physical performance in CKD patients not yet on dialysis might provide valuable data on health-related risks, and result in improved care, if the causes of declined exercise capacity are better comprehended.

We aimed to study maximal physical performance and its determinants including cardiac biomarkers, AAC and echocardiography in patients with stage 4–5 CKD not on maintenance dialysis.

## Methods

### Study protocol

The Chronic Arterial Disease, quality of life and mortality in chronic KIDney injury (CADKID) -study (http://www.ClinicalTrials.gov NCT04223726) is an ongoing prospective follow-up study protocol assessing arterial disease, quality of life and mortality in patients with CKD stage 4–5. Two hundred ten consecutive patients referred to the predialysis outpatient clinic of Kidney Center, Turku University Hospital between 2013 and 2017 were recruited to the study. Inclusion criteria for the study participants were ≥ 18 years of age and CKD stage 4–5 with an estimated glomerular filtration rate (eGFR) < 30 ml/min per 1.73 m2 calculated using the Chronic Kidney Disease Epidemiology Collaboration (CKD-EPI) equation [16]. The study design was approved by Medical Ethics Committee of the Hospital District of Southwest Finland. All procedures were in accordance with the Helsinki Declaration. All patients gave written informed consent before entering the study.

This is a pre-specified preliminary report of the CADKID study on the factors associated with baseline stress ergometry performance. Of all CADKID study patients 194 were invited to attend stress ergometry in the beginning of the study. In 16 study patients the stress ergometry was deferred due to severe disability, illness or contraindication. One hundred eighty patients agreed to attend, but the ergometry was cancelled in 3 patients who started maintenance dialysis prior to ergometry testing and in 3 patients stress ergometry was considered contraindicated on the morning of the study. Therefore 174 patients underwent a standard maximal bicycle stress test (Supplemental Figure 1). Abdominal aortic calcification was assessed from plain lateral lumbar radiograph, and echocardiography in addition to biochemical studies were examined at baseline.

### Maximal stress ergometry

Maximal Stress Ergometry was performed as an incremental, symptom-limited cycling exercise test in accordance with clinical standards. Each patient started with a 30s warm-up phase during which the target speed of 60 rpm was reached. Primary workload was determined according to an estimated maximum workload and a targeted test duration of 6 to 10 min. An increase in workload per minute (10, 15 or 20 W) was accomplished automatically by the ergometer software until symptom limitation within 6 to 10 min. Participants were informed to cycle at a speed of 60 rpm, and were encouraged to continue cycling until exhaustion. Perceived strain was reported as the highest rating on the Borg Scale from 1 to 20. The mean proportional workload of the last 4 min of the age, sex and body size predicted value (WMAX%), was used in the analyses in addition to the corresponding workload in watts (WMAX). The values considered normal for expected maximal exercise performance measured as watts are derived and extrapolated from large data sets of the Mini Suomi –study [17]. The algorithm for expected exercise performance is incorporated in cycle ergometer software and used in day-to-day clinical work. The study population was divided into two groups according to WMAX% < 50 versus ≥50% of the expected normal value.

### Echocardiography

A comprehensive echocardiographic examination was performed at rest before the exercise test at the Department of Clinical Physiology of Turku University Hospital. Data collected included the systolic and diastolic dimensions and function of the left ventricle (LV), left ventricular wall thickness, aortic and left atrial dimensions, LV mass index (LVMI), LV ejection fraction (LVEF), global longitudinal strain (GLS), and early maximal ventricular filling velocity and the late filling velocity (E/A-ratio). For E/e’ the transmitral early diastolic inflow velocity (E wave) was measured using pulse-wave Doppler in the apical four-chamber view, and the peak (e’) diastolic mitral annular velocity was measured using tissue Doppler imaging at the septal mitral annulus. Ultrasound examinations were performed using a commercially available ultrasound system (Vivid E9; GE Vingmed Ultrasound, Horten, Norway) with a 3.5-MHz phased-array transducer (M5S).

### Assessment of AAC

Abdominal aortic calcification (AAC) score was calculated for each subject. Lateral lumbar radiography with standard equipment was performed in a standing position. A validated 24-point scale, as described previously, was used [18]. Calcific deposits of the anterior and posterior wall of the aorta, adjacent to first through fourth lumbar vertebrae, were assessed at each vertebral segment, and were graded on a scale 0–3 as follows: 0 = no calcific deposits, 1 = small scattered calcific deposits filling less than one-third of the longitudinal aortic wall, 2 = one-thirds to two-thirds of the longitudinal aortic wall calcified, 3 = at least two-thirds of the longitudinal aortic wall calcified. The grades of eight segments were summed ranging from 0 to 24 points (Supplemental Figure 2). Two independent observers recorded AAC scores of all lateral lumbar X-rays, and mean was used for analysis.

### Statistical analysis

Results are presented as mean ± standard deviation (SD) for the normally distributed variables and as median [inter-quartile range (IQR)] for skewed variables. Skewed variables were log_e_-transformed to normalize distributions. Normality in continuous covariates was tested with Kolmogorov-Smirnov and Shapiro-Wilk tests. Student’s t-test was used to compare continuous normally distributed covariates and Chi-square test for categorical covariates in the study subgroups. For some skewed variables a suitable transformation was not found and thus the comparisons between groups were done using a non-parametric Kruskal-Wallis test.

Univariable associations between the study variables were analyzed by calculating Spearman’s correlation coefficients. Multivariable analysis was done using linear regression technique. Potential existence of multicollinearity was assessed by examining variance inflation factors. Variables with significant univariable correlations with WMAX% (diastolic blood pressure, Troponin T, proBNP, hemoglobin, leukocytes, AAC, E/e’-ratio and GLS), as well as diabetes and previous coronary artery disease were included as covariates in stepwise multivariable linear regression models. The multivariable associations between exposure variables and WMAX were also studied using the similar analyses. However, for WMAX (expressed in Watts), age, height and gender were also included as covariates in the initial stepwise multivariable model. To make the multivariable results easily comparable, variables were included in the final models without transformation.

Receiver operating characteristics (ROC) curve analyses were conducted to estimate the area under the curve (AUC) as a measure of discriminative capacity of TnT and AAC for WMAX% < 50%. Generally, we consider an AUC > 0.90 outstanding, an AUC 0.80–0.90 excellent, an AUC 0.70–0.80 acceptable and an AUC < 0.70 poor discrimination [19].

All statistical analyses were performed using statistical analysis system, SAS version 9.3 (SAS Institute Inc., Cary NC). *P* < 0.05 was considered statistically significant.

## Results

### Patient characteristics

A total of 174 patients with a mean age of 60.9±13.7 years and median eGFR 12.9 ± 3.4 ml/min/1.73 m^2^ underwent a maximal bicycle stress test, echocardiography and lateral lumbar radiography for AAC assessment. Nearly half (43) had diabetes and 21 (12) had coronary artery disease. All, but one were on antihypertensive medication. The mean workload of the last 4 minutes of maximal stress (WMAX) was 83.7 ± 36.5 W and the proportional maximal workload (WMAX%) 55.7 ± 21.5% of the age predicted normal value. The baseline clinical and laboratory characteristics of the study groups (WMAX% < 50 vs. ≥50%) are shown in Table 1.

**Table 1:** Groupwise comparisons according to WMAX%. Values are presented as mean ± SD for normally distributed variables and median (IQR) for skewed variables

Variable	Maximal workload < 50% of expected normal	≥50% of expected normal	*P*-value
Number of subjects, n	71	103	–
**Demographics and medications**			
Female subjects, n (%)	14 (20)	40 (39)	0.007
Age (years)	64.5±13.2	58.5±13.7	0.005
Diabetes, n (%)	44 (62)	31 (30)	< 0.0001
Coronary artery disease, n (%)	14 (20)	7 (7)	0.01
Beta blockers, n (%)	57 (80)	71 (69)	0.10
Calcium channel blockers, n (%)	59 (83)	81 (79)	0.47
Body mass index (kg/m^2^)	28.6±6.7	27.7±4.6	0.33
**Physiological and stress ergometry data**			
Systolic blood pressure (mmHg)	154 (139–167)	148 (136–162)	0.52
Diastolic blood pressure (mmHg)	78 ± 15	85 ± 12	0.0005
Pulse pressure (mmHg)	73 (59–87)	65 (51–76)	0.01
WMAX (W)	55 ± 21	104 ± 31	< 0.0001
MET (units)	3.5 (3.1–4.2)	5.3 (4.7–6.6)	< 0.0001
Maximum heart rate (1/min)	104 (93–117)	136 (114–148)	< 0.0001
**Biochemical data**			
Creatinine (μmol/l)	420 ± 105	408±97	0.53
eGFR (ml/min)	12 (10–15)	12 (11–15)	0.40
Urea (mmol/l)	23.9 ± 6.6	21.9±5.7	0.04
Hemoglobin (g/l)	113 ± 12	116±13	0.14
C-reactive protein (mg/l)	2 (1–5)	2 (1–4)	0.11
Albumin (g/l)	34 (31.6–36.8)	35.9 (32.8–38.4)	0.06
Sodium (mmol/l)	142 (140–144)	141 (140–143)	0.47
Potassium (mmol/l)	4.4 (4.0–4.6)	4.4 (4.0–4.7)	0.68
Ionized calcium (mmol/l)	1.19 (1.14–1.23)	1.20 (1.18–1.24)	0.07
Phosphorus (mmol/l)	1.51 (1.27–1.70)	1.37 (1.26–1.62)	0.03
Parathyroid hormone (ng/l)	185 (121–367)	180 (127–279)	0.37
Troponin T (ng/l)	49 (29.5–76.0)	25.0 (16.5–25.0)	< 0.0001
CK-MB mass (μg/l)	2.6 (1.8–3.6)	2.35 (1.50–3.75)	0.26
proBNP (ng/l)	1915 (888–4485)	722 (362–1510)	< 0.0001
pH	7.39 (7.37–7.41)	7.38 (7.35–7.41)	0.19
Bicarbonate (mmol/l)	22.5 ± 2.7	22.3 ± 2.5	0.61
Total cholesterol (mmol/l)	4.3 (3.5–4.8)	4.5 (3.7–5.2)	0.13
HbA1c (%)	5.8 (5.3–6.7)	5.2 (5.0–5.9)	0.003
**Echocardiography and AAC**			
LV ejection fraction (%)	65 (59–70)	65 (61–69)	0.72
LV end diastolic diameter (mm)	56 (50–59)	53 (50–56)	0.03
LV mass index	114 (96–136)	97 (85–117)	0.0001
E/e’ *n* = 124	10.0 (8.9–13.0)	8.4 (7.0–11.0)	0.0008
GLS *n* = 123	17.4 (14.5–19.2)	19.15 (17.0–20.6)	0.0005
AAC score	8.5 (3.5–13.0)	3.0 (0.5–7.5)	< 0.0001

*WMAX%* The mean proportional workload of the last 4 min of the age, sex, and body size predicted value, *WMAX* Mean workload of the last four minutes of maximal stress, *MET* metabolic equivalent of task, *eGFR* Estimated glomerular filtration rate, *CK-MB* creatine kinase-isoenzyme MB, *proBNP* N-terminal pro-B-type natriuretic peptide, *AAC* Abdominal Aortic Calcification, *LV* left ventricular, *E/e’* ratio of transmitral Doppler early filling velocity to tissue Doppler early diastolic mitral annular velocity, *GLS* Left ventricular global longitudinal strain, *AAC* Abdominal Aortic Calcification

Data are presented only for the 174 CADKID study patients that underwent stress ergometry. We compared the patients not attending stress ergometry (*n* = 36) to those who did (*n* = 174). Those not attending were more often women, had a higher proportion of coronary artery disease (CAD), were older and had higher TnT but no differences were observed in AAC or proportion of diabetics (Supplemental Table 1).

### Determinants of exercise performance

The subjects with WMAX% of less than 50% of the expected values were older, more likely men, and had a higher prevalence of diabetes and coronary artery disease. TnT, proBNP, AAC, left ventricular end-diastolic diameter, LVMI, E/e’, and pulse pressure were higher compared to the better performing group and GLS was lower. There were no significant differences between the groups in terms of body mass index, eGFR, hemoglobin, albumin, total cholesterol, LVEF or the use of beta blockers or calcium channel blockers. Only 5 patients in the whole cohort had below normal (< 50%) LVEF.

Univariable correlates of relative exercise performance are shown in Table 2. TnT, proBNP, E/e’, LVMI, AAC, pulse pressure, leukocytes, ESR and CRP were negatively and GLS was positively correlated with WMAX%. Patients with diabetes and CAD had significantly lower WMAX% compared to those without these conditions (No diabetes or CAD: 65.2 ± 21.0%, Diabetes: 45.6 ± 15.7%, CAD: 42.5 ± 18.3%, *p* < 0.0001 for both comparisons).

**Table 2:** Univariable correlates of WMAX%

Variable	Correlation coefficient	*P*-value
Troponin T	−0.52	< 0.0001
proBNP	−0.39	< 0.0001
AAC	−0.46	< 0.0001
E/e’	−0.41	< 0.0001
LVMI	−0.25	0.001
GLS	0.27	0.002
Diastolic blood pressure	0.35	< 0.0001
Pulse pressure	−0.34	< 0.0001
Hemoglobin	0.17	0.02
Leukocytes	−0.35	< 0.0001
Erythrocyte sedimentation rate	−0.25	0.0009
C-reactive protein	−0.22	0.003

*proBNP* N-terminal pro-B-type natriuretic peptide, *AAC* Abdominal Aortic Calcification, *E/e’* ratio of transmitral Doppler early filling velocity to tissue Doppler early diastolic mitral annular velocity, *LVMI* Left ventricular mass index, *GLS* Left ventricular global longitudinal strain

TnT (β = − 0.09, *p* = 0.02), AAC (β = − 1.67, *p* < 0.0001) and diabetes (β = − 11.7, *p* < 0.0001) remained as significant predictors for WMAX% in the stepwise multivariable linear regression model. When WMAX (in Watts) was included as the dependent variable in the multivariable model (instead of WMAX%) the significant explanatory variables were TnT (β = − 0.13, *p* = 0.046), AAC (β = − 1.44, *p* = 0.001), age (β = − 0.97, *p* < 0.0001), male gender (β = 31.0, *p* < 0.0001), hemoglobin (β = 0.42, *p* = 0.01), leukocytes (β = − 2.09, *p* = 0.046) and diastolic blood pressure (β = 0.30, *p* = 0.05) and diabetes (β = 16.1, *p* = 0.0005) (Table 3).

**Table 3:** Stepwise multivariable analyses for WMAX and WMAX% as dependent variables

Variables in the final model (significance < 0.15)	β	*P*-value
Stepwise multivariable model for WMAX		
AAC score	−1.44	0.001
Diabetes	−16.06	0.0005
Troponin T	− 0.13	0.046
Leukocytes	−2.09	0.046
Hemoglobin	0.42	0.01
Diastolic blood pressure	0.30	0.05
Male gender	31.00	< 0.0001
Age	−0.97	< 0.0001
Stepwise multivariable model for WMAX%		
AAC score	−1.67	< 0.0001
Diabetes	− 11.7	< 0.0001
Troponin T	−0.09	0.02
Leukocytes	−1.47	0.08

Exposure variables included in the initial stepwise model for WMAX%: Diabetes; Coronary artery disease; Diastolic blood pressure; Troponin T; *proBNP* N-terminal pro-B-type natriuretic peptide; Hemoglobin; Leukocytes; *AAC* Abdominal Aortic Calcification score, *E/e’* ratio of transmitral early filling velocity, *GLS* Left ventricular global longitudinal strain. Exposure variables included in the initial stepwise model for WMAX: The exposure variables for WMAX% in addition to gender, age and height

**Fig. 1:**
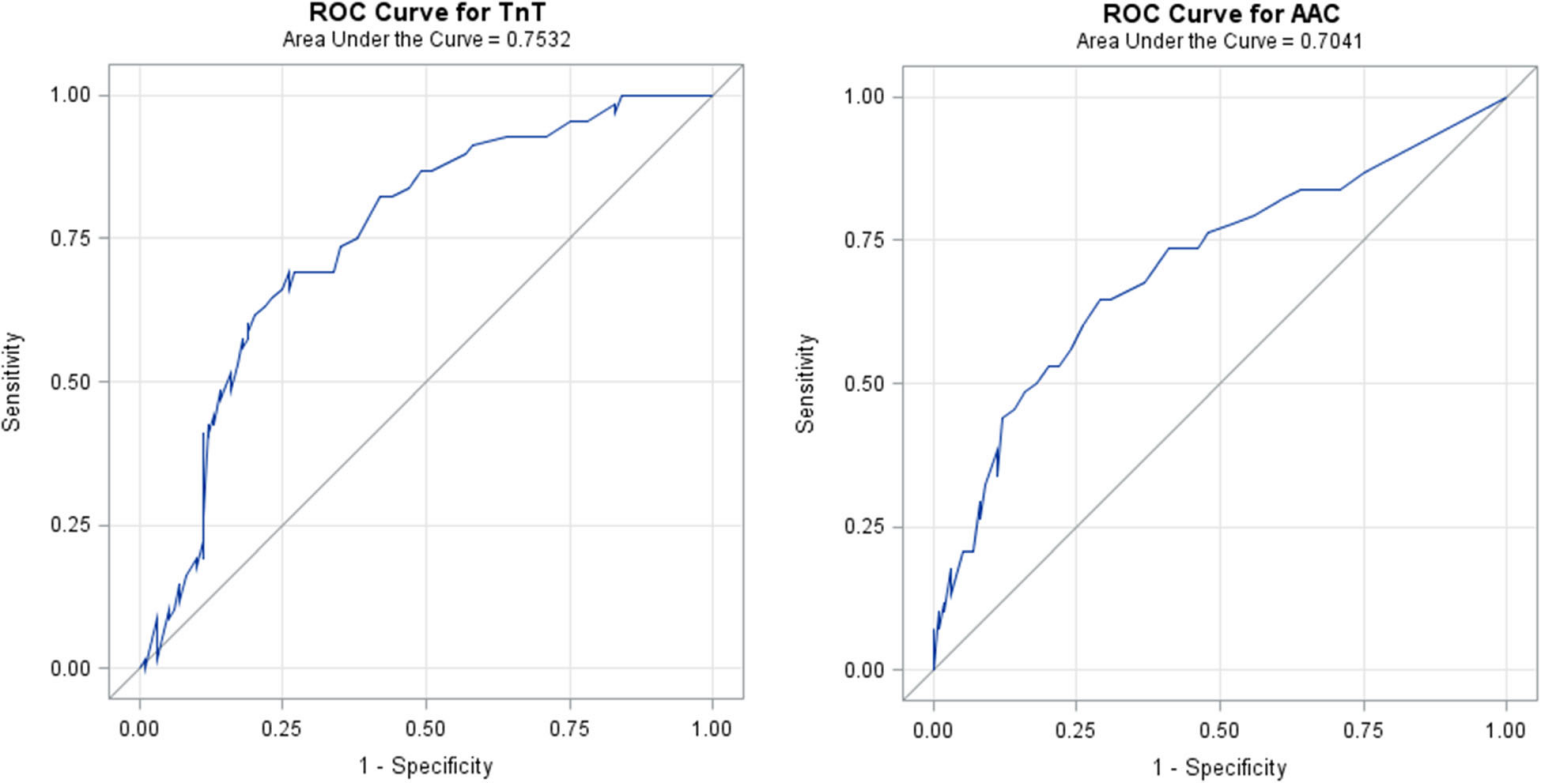
Area under the curve (AUC) of receiver operating characteristics curve (ROC) analyses for Troponin T (TnT) (left panel) an abdominal aortic calcification (AAC) score (right panel) in relation to maximal proportional stress ergometry workload of less than 50% of the expected normal value

TnT was associated with LVMI (*r* = 0.34, *p <* 0.0001), E/e’ (r = 0.28, *p* = 0.002), GLS (*r* = − 0.27, *p* = 0.003) and creatinine (*r* = 0.20, *p* = 0.01) but not with LVEF or eGFR. AAC was significantly associated with E/e’ (*r* = 0.48, *p* < 0.0001). TnT and AAC showed fair predictive power for WMAX% less than 50% in ROC curve analyses. TnT exhibited an AUC of 0.75 (95%CI 0.68–0.83) and AAC an AUC of 0.70 (95%CI 0.62–0.79) (Fig. 1).

## Discussion

The present study shows for the first time that maximal stress ergometry performance is associated with TnT and AAC but not with proBNP in CKD stage 4–5 patients without maintenance dialysis.

Previous studies have demonstrated that elevated TnT is associated with an increased risk for incident cardiovascular events and mortality in CKD [4–6]. The Food and Drug Administration and the National Kidney Foundation Disease Outcomes Quality Initiative Work Group (KDOQI) have recommended that TnT levels can be considered for risk stratification of CKD patients to identify those at high mortality risk [5, 20]. Our current results suggest that increased TnT in CKD is a determinant of cardiovascular burden that affects the day to day life of patients with advanced CKD by limiting their maximal physical performance in addition to increasing their risk for incident cardiovascular death. In accordance with this finding we have previously shown that the physical composite score of the Short Form 36 Items Health Survey (SF-36 QOL) is inversely associated with TnT in the CADKID study population [21].

A recent large study showed that increased TnT is associated with left ventricular hypertrophy (LVH) and diastolic dysfunction in CKD but not with systolic dysfunction [22]. In line with these results TnT was associated with left ventricular mass index, E/e’ and GLS but not with LVEF in present study. Only 5 patients in our study cohort had below normal LVEF (< 50%) which may explain why proBNP was not independently associated with WMAX% in the multivariate model. Increased E/e’–ratio, a marker of cardiac diastolic dysfunction, caused mainly by fluid and sodium retention and ventricular stiffness in CKD, showed significant univariable correlations with both TnT and WMAX% as did GLS, a more subtle indicator of left ventricular function compared to LVEF. These alterations in cardiac function probably have a significant role in the attenuated physical performance in CKD. However, none of the associations between WMAX% and echocardiographic indices remained significant in the multivariate model in comparison to TnT which remained highly significant. Therefore, increased TnT may at least to some extent be a marker of silent myocardial ischemia in our patients with advanced CKD which could explain the reduced ergometry performance in affected patients. In line with this assumption a previous single photon emission tomography study with mostly maintenance dialysis dependent ESKD patients showed an association between TnT and perfusion defects indicative of myocardial ischemia after pharmacologic and/or exercise stress [23]. The association between TnT and WMAX% also remained significant after controlling for previously diagnosed CAD in the multivariable model.

AAC is independently associated with cardiovascular events in the general population and in dialysis patients and the KDOQI Work Group recommends the assessment of AAC for risk stratification in CKD [10–12]. There are no previous data available on the association between AAC and maximal ergometry performance or other physical stress tests in patients with CKD. AAC has previously been shown to be associated with left ventricular mass, left atrial volume and left ventricular diastolic dysfunction including decreased E/e’ in CKD [14, 24]. Furthermore, former studies have shown that AAC is independently associated with coronary artery calcification [13], and increased risk for incident claudication [15], both of which may lead to diminished physical performance in CKD. In line with previous findings AAC was associated with E/e’ in addition to attenuated exercise capacity in the present study. As AAC is closely associated with coronary artery calcification the association with poor maximal exercise tolerance may be at least partly attributed to silent myocardial ischaemia during the maximal ergometry test. Other potential mechanisms responsible for the observed attenuation of maximal stress test workload with increasing AAC in our study include universal atherosclerosis and peripheral arterial disease and increased ventricular stiffness and diastolic dysfunction that are known to be associated with aortic calcification. The finding that AAC has an impact not only for risk stratification of patients with advanced CKD, but also on their ability to endure exercise, may be of value in clinical work to target treatments and further diagnostics accordingly.

The present study has limitations. The data included in this current report are cross-sectional and from a single center which may increase the possibility of residual confounders and limit the generalizability of the findings. The study sample was somewhat limited. There was a degree of expected selection bias in attending stress ergometry as the patients not attending were more often women and had CAD, were older and had higher TnT but no differences were observed in AAC or proportion of diabetics (Supplemental Table 1). Nevertheless, in our opinion the studied cohort with a high degree of comorbidities represents the overall CKD stage 4–5 population at our center well. It is not likely that our current results on the association between TnT, AAC and WMAX were significantly affected due to this selection bias. As we did not perform spiroergometry to define the peak oxygen uptake in this study, the maximal ergometry performance was therefore limited by subjective exhaustion. However, we believe the results give a reliable estimate of maximal aerobic performance as the observed relative value of 55.7 ± 21.5% of the age predicted performance was similar to the reduction in maximal oxygen uptake reported in patients on maintenance dialysis [25]. Previous cross-sectional data from two large cohorts have shown that urinary albumin/creatinine –ratio is inversely associated with physical activity [26]. Unfortunately urinary albumin/creatinine –ratio was not included in the CADKID study protocol and we could not examine its association with WMAX. A high proportion of patients were on beta blockers and the ergometry was performed without medication pauses, which has probably influenced the workloads achieved in affected patients. However, beta blocker use was similar between patients with WMAX% < 50 compared to others.

CKD is associated with inferior exercise capacity and the decrease in physical function appears to emerge in the predialysis period [27]. Maintaining functional independence is of most importance in patient-reported questionnaires filled by CKD patients [28]. The recent prospective randomized multicenter EXCITE trial showed that a personalized walking exercise program at home improves the physical performance of dialysis patients and reduces their rate of hospitalization [29]. The finding that TnT and AAC are independently associated with maximal physical performance in advanced CKD may have clinical implications in recognizing patients at risk and targeting treatment not only for decreasing mortality risk but also to increase the functionality and quality of life of affected patients.

## Conclusion

Our current findings show for the first time that TnT and AAC are independently associated with maximal ergometry stress test workload in patients with advanced CKD not on dialysis.

## Supplementary Information

**Additional file 1: Supplemental Figure 1**. Flow chart of the study.

**Additional file 2: Supplemental Figure 2**. Examples of abdominal aortic calcification (AAC) score assessment in plain lateral lumbar radiograph. AAC score is the sum of scores of anterior and posterior wall calcific deposits of L1 through L4 shown in the figure. Severe AAC (AAC score 20/24) in a > 60-year-old male with CKD stage 5 (Panel A) and no aortic calcific deposits (AAC score 0/24) in a < 40-year-old female with CKD stage 5 (Panel B). L = lumbar vertebra.

**Additional file 3: Supplemental Table 1**. Comparison between patients included in the study and patients excluded due to missing stress ergometry data.

## Data Availability

Data that support the findings of this study are available from the datasets of the Kidney Center of Turku University Hospital upon reasonable request from the corresponding author and after permission of the Ethics Committee from Hospital District of Southwest Finland.
