# Supplementary figures and images for: Association of maximal stress ergometry performance with troponin T and abdominal aortic calcification score in advanced chronic kidney disease
Source: BMC Nephrol. 2021 Feb 4;22:50. doi: 10.1186/s12882-021-02251-y (PMC7863467; doi:10.1186/s12882-021-02251-y)

## Slide 1
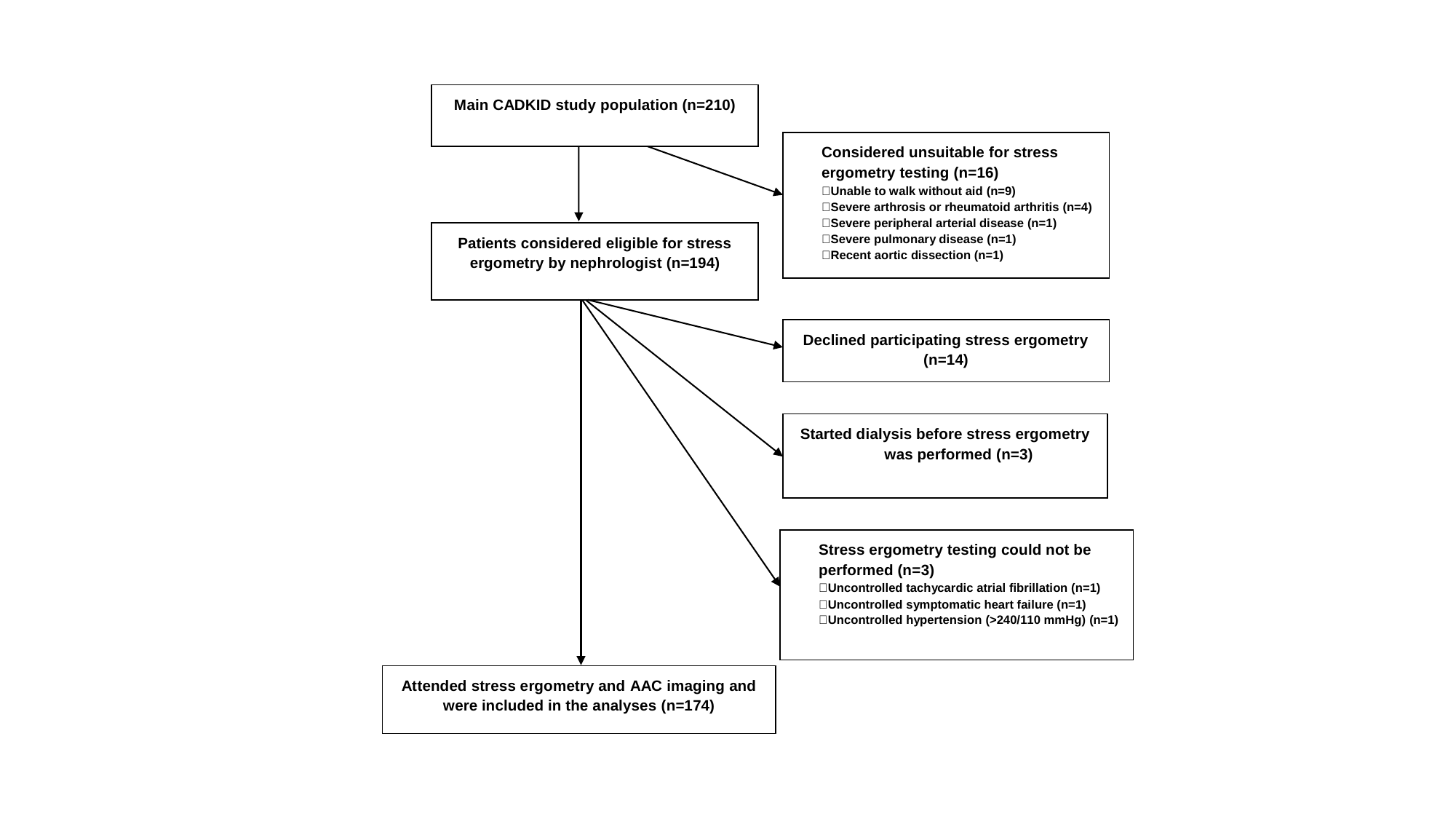

Supplement: Supplementary file 1 — Additional file 1: Supplemental Figure 1. Flow chart of the study. [file 12882_2021_2251_MOESM1_ESM.pptx]

## Slide 1
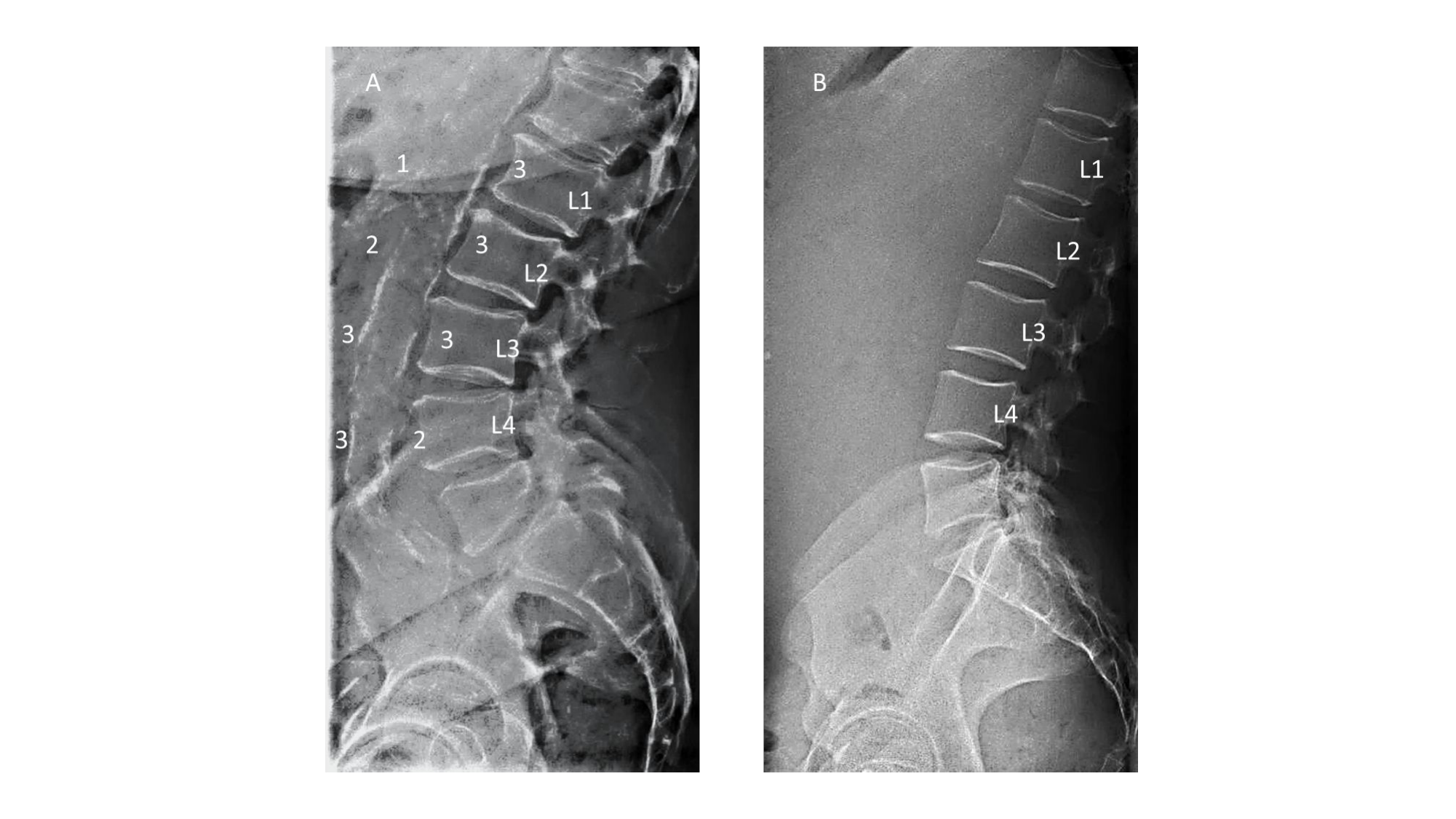

B
A
1
3
L1
L1
2
3
L2
L2
L3
3
3
L3
L4
L4
3
2

Supplement: Supplementary file 2 — Additional file 2: Supplemental Figure 2. Examples of abdominal aortic calcification (AAC) score assessment in plain lateral lumbar radiograph. AAC score is the sum of scores of anterior and posterior wall calcific deposits of L1 through L4 shown in the figure. Severe AAC (AAC score 20/24) in a > 60-year-old male with CKD stage 5 (Panel A) and no aortic calcific deposits (AAC score 0/24) in a < 40-year-old female with CKD stage 5 (Panel B). L = lumbar vertebra. [file 12882_2021_2251_MOESM2_ESM.pptx]
