# Supplementary material for: Association of maximal stress ergometry performance with troponin T and abdominal aortic calcification score in advanced chronic kidney disease
Source: BMC Nephrol. 2021 Feb 4;22:50. doi: 10.1186/s12882-021-02251-y (PMC7863467; doi:10.1186/s12882-021-02251-y)
Supplement: Supplementary file 3 — Additional file 3: Supplemental Table 1. Comparison between patients included in the study and patients excluded due to missing stress ergometry data. [file 12882_2021_2251_MOESM3_ESM.docx]

Supplemental Table 1. Comparison between patients included in the study and patients excluded due to missing stress ergometry data

| Variable | Included in the study (n=174) | Excluded due to missing ergometry (n=36) | *P*-value |
| --- | --- | --- | --- |
| Age (years) | 61±14 | 70±12 | 0.0004 |
| Women, n (%) | 54 (31) | 19 (53) | 0.01 |
| Coronary artery disease, n (%) | 21 (12) | 13 (36) | 0.0004 |
| Diabetes, n (%) | 75 (43) | 19 (53) | 0.29 |
| eGFR (ml/min) | 12 (11-15) | 12 (10-15) | 0.50 |
| Hemoglobin (g/l) | 115±12 | 111±13 | 0.14 |
| Troponin T (ng/l) | 31 (21-56) | 64 (46-116) | <0.0001 |
| proBNP (ng/l) | 1030 (450-2560) | 2645 (1300-8950) | 0.0008 |
| AAC score | 5.5 (1.0-10.5) | 8.0 (2.0-12.0), n=25 | 0.22 |

eGFR = Estimated glomerular filtration rate; proBNP = N-terminal pro-B-type natriuretic peptide; AAC=Abdominal Aortic Calcification
